# Growth performance and total tract digestibility of nutrients for weanling pigs are improved by an exogenous xylanase and a stimbiotic regardless of maternal xylanase consumption

**DOI:** 10.1186/s40104-025-01205-w

**Published:** 2025-05-15

**Authors:** Jessica P. Acosta, Charmaine D. Espinosa, Gemma González-Ortiz, Hans H. Stein

**Affiliations:** 1 Division of Nutritional Sciences, University of Illinois, Urbana, IL 61801 USA; 2 Department of Animal Sciences, University of Illinois, Urbana, IL 61801 USA; 3 EnviroFlight, Raleigh, NC USA; 4 AB Vista, Marlborough, SN8 4AN UK

**Keywords:** Digestibility, Growth performance, Sows, Stimbiotic, Weanling pigs, Xylanase

## Abstract

**Background:**

Exogenous xylanase can increase utilization of fiber and energy when included in diets for pigs, and xylo-oligosaccharides (XOS) may improve growth performance of pigs by modulating intestinal fermentation. However, it is unclear if a stimbiotic (i.e., a combination of xylanase and XOS) has superior effects compared with a xylanase alone, and there is a lack of data demonstrating if xylanase fed to lactating sows influences growth performance of weanling pigs. Therefore, two hypotheses were tested: 1) xylanase and stimbiotic improve growth performance, apparent total tract digestibility (ATTD) of gross energy (GE) and total dietary fiber (TDF), digestible energy (DE), and intestinal health of weanling pigs and 2) offspring of sows fed xylanase in lactation have greater growth performance after weaning than offspring of sows fed no xylanase during lactation.

**Methods:**

One hundred and twenty pigs were weaned from sows fed a diet without xylanase, and 120 pigs were weaned from sows fed a lactation diet containing 16,000 beechwood xylanase units per kg (initial weight: 5.81 ± 0.50 kg). Pigs were allotted to a 2 × 3 factorial with two sow groups (lactation diet without or with xylanase) and three dietary treatments (i.e., control, control plus xylanase, or control plus stimbiotic).

**Results:**

There were no interactions between sow treatment and post-weaning pig treatment, and sow treatment did not impact post-weaning growth or ATTD of GE and TDF in weaned pigs. From d 15 to 28 post-weaning, the ADG, G:F, ATTD of GE and TDF, and concentration of DE were greater (*P* < 0.05) for pigs fed the diet with stimbiotic than if fed the xylanase diet or the control diet, and pigs fed the xylanase diet had greater (*P* < 0.05) ADG, G:F, ATTD of GE and TDF, and concentration of DE than pigs fed the control diet. From d 29 to 42 post-weaning, pigs fed the diets with xylanase or stimbiotic had greater (*P* < 0.05) ADG, ATTD of GE and TDF, and DE than pigs fed the control diet.

**Conclusions:**

Pigs fed xylanase or stimbiotic had greater ATTD of GE and TDF, greater DE, and greater overall ADG, G:F, and final body weight on d 42 post-weaning than pigs fed the control diet, but feeding sows xylanase in lactation did not influence post-weaning growth performance.

**Supplementary Information:**

The online version contains supplementary material available at 10.1186/s40104-025-01205-w.

## Background

Arabinoxylans, a polysaccharide made up of a chain of xylose units with sidechains of arabinose, galactose, and acetyl group, make up the majority of the fiber in cereal grains and cereal co-products [1]. Arabinoxylans cannot be hydrolyzed by endogenous enzymes, thus they pass through the small intestine undigested, but interfere with nutrient digestibility, water absorption, and digesta passage time [2]. Exogenous xylanase hydrolyzes the β-(1−4) glycosidic bonds between xylose units in the backbone of arabinoxylan, which results in the production in situ of arabinoxylo-oligosaccharides (AXOS). Xylo-oligosaccharides (XOS) and AXOS may be fermented by intestinal microbes in the hindgut to release short-chain fatty acids that can provide energy to the animals [3]. Because of the stimulation of fermentation that is initiated by xylanase, utilization of dietary fiber and energy may be increased if xylanase is included in the diet, resulting in greater growth performance and improved gut barrier integrity of weanling pigs [4–7]. Xylo-oligosaccharides and AXOS are carbohydrates made up of 2 to 10 xylose units linked through β-(1→4)-linkages and sidechains of arabinose units or acetyl groups [8, 9]. Xylo-oligosaccharides may improve growth performance of weanling pigs because they can modulate the gut microbiota at very low doses [10–13]. A stimbiotic (i.e., the combination of xylanase and XOS) may also improve growth performance of weanling pigs by shifting the intestinal microbiome to favor fermentation of fiber [14], but it is not known if the effect of a stimbiotic is greater than that of xylanase.

Feeding xylanase to lactating sows may result in increased energy and nutrient digestibility [15, 16], and possibly also a change in intestinal microbiome, which may influence the microbiome of nursing pigs. It is, therefore, possible that feeding xylanase to lactating sows results in a carry-over effect on growth performance, digestibility of nutrients, and intestinal health of weanling pigs, but this hypothesis has not been investigated. Therefore, an experiment was conducted to test the hypothesis that xylanase alone or a stimbiotic improves growth performance and the apparent total tract digestibility (ATTD) of dry matter, gross energy (GE), crude protein, and total dietary fiber (TDF), and the concentration of digestible energy (DE) of diets, and intestinal health of weanling pigs. The second hypothesis was that the effect of using xylanase in post-weaning diets is greater in offspring of sows fed xylanase in lactation than in offspring of sows that were not fed xylanase during lactation.

## Methods

The protocol for the experiment was submitted to the Institutional Animal Care and Use Committee at the University of Illinois and was reviewed and approved prior to initiation of the experiment. Pigs used in this experiment were the offspring of Camborough sows mated to Line 800 boars (Pig Improvement Company, Hendersonville, TN, USA).

### Animals, housing, experimental design and diets

One hundred and twenty pigs were weaned from sows fed a control diet without xylanase and 120 pigs were weaned from sows fed a diet containing 16,000 beechwood xylanase units (BXU) per kg of an exogenous xylanase (240 weaned pigs in total; initial body weight: 5.81 ± 0.50 kg). One beechwood xylanase unit is defined as the amount of enzyme that will release 0.06 micromoles of reducing sugars (xylose equivalents) from beechwood xylan per min at pH 5.3 and 50 °C. Sows had been fed experimental diets for two reproductive cycles and pigs used in this experiment were weaned at the end of the second cycle. Details of sow treatments and performance were published elsewhere [15]. Pigs were weaned in 4 blocks of 60 pigs per block, and weaning group was used as the blocking factor. Pigs in each block were placed in a separate weaning units; thus there were a total of 4 weaning barns used in the experiment. The temperature in the barns was 30 °C in week one after weaning, 28 °C in week 2 after weaning, the temperature was then reduced by 1 °C in each of the following weeks post-weaning. Pigs were housed in mixed-sex pens in groups of 5 pigs per pen, and sex was balanced among treatments. There were 6 treatments and 8 replicate pens per treatment. Pens had fully slatted floors, a feeder, and a nipple drinker. The experimental design was a 2 × 3 factorial with two sow treatments (sows fed the diet without xylanase and sows fed the diet with xylanase) and three dietary treatments after weaning (i.e., control, control plus 100 g/t of xylanase, or control plus 100 g/t of stimbiotic). Xylanase (Econase XT 25; AB Vista, Marlborough, UK) and stimbiotic (Signis; AB Vista, Marlborough, UK) were included in diets for weanling pigs as recommended by the supplier. The 100 g/t of xylanase in the diets was expected to provided 16,000 BXU of xylanase in the xylanase and the stimbiotic treatments. A 3-phase feeding program was used. Days 1 to 14 were phase 1, d 15 to 28 were phase 2, and d 29 to 42 were phase 3. Diets were based on corn, soybean meal, and wheat middlings, and phase 1 and phase 2 diets also contained fermented soybean meal (Industrial De Oleaginosas Americanas S.A., Barranca, Costa Rica) and lactose, and phase 1 diets contained fish meal as well (Table 1). Pigs were fed experimental diets in all three phases (Tables 2 and 3). All diets were formulated to meet nutrient requirements for weanling pigs [17] and all diets were fed as mash. Titanium dioxide (0.4%) was included in all diets as an indigestible marker. Pigs were allowed ad libitum intake of feed and water throughout the experiment.

**Table 1:** Analyzed nutrient composition of the main ingredients in diets (as-fed basis)

Item	Corn	Soybean meal	Wheat middlings	Fermented soybean meal^a^	Fish meal
Gross energy, kcal/kg	3,816	4,133	3,997	4,213	4,320
Dry matter, %	85.85	89.25	88.88	87.63	94.93
Ash, %	1.33	6.12	5.99	6.69	20.82
Acid hydrolyzed ether extract, %	3.70	3.30	4.84	0.75	10.15
Crude protein, %	6.58	47.25	15.37	49.1	64.03
Starch, %	65.40	1.27	19.70	1.35	-
Insoluble dietary fiber, %	9.20	14.30	38.40	15.90	3.20
Soluble dietary fiber, %	0.70	3.70	3.20	3.70	1.30
Total dietary fiber, %	9.90	18.00	41.60	19.60	4.50
Indispensable amino acids, %					
Arg	0.31	3.29	1.05	3.14	3.67
His	0.19	1.22	0.42	1.21	1.33
Ile	0.26	2.21	0.51	2.42	2.53
Leu	0.76	3.61	0.91	3.74	4.17
Lys	0.24	2.95	0.65	2.92	4.79
Met	0.13	0.65	0.21	0.65	1.63
Phe	0.32	2.41	0.58	2.50	2.33
Thr	0.23	1.82	0.47	1.87	2.44
Trp	0.04	0.55	0.15	0.66	0.52
Val	0.32	2.37	0.72	2.46	2.89
Dispensable amino acids, %					
Ala	0.48	2.03	0.71	2.16	3.90
Asp	0.44	5.21	1.06	5.46	5.44
Cys	0.15	0.66	0.33	0.67	0.49
Glu	1.21	8.57	2.74	8.35	8.03
Gly	0.27	1.95	0.81	2.06	4.74
Pro	0.56	2.28	0.90	2.44	2.98
Ser	0.29	2.04	0.54	2.07	2.09
Tyr	0.20	1.68	0.37	1.70	1.75
Minerals					
Ca, %	0.02	0.33	0.20	0.33	5.39
P, %	0.25	0.71	0.99	0.75	3.53
K, %	0.30	2.61	1.13	2.29	1.14
Mg, %	0.07	0.33	0.40	0.29	0.25
Na, %	0.01	0.15	0.02	0.01	0.77
Cu, mg/kg	6.87	43.50	34.05	37.94	66.11
Fe, mg/kg	33.45	210.86	154.52	123.22	1,058.61
Mn, mg/kg	12.56	86.90	169.01	76.54	177.22
Zn, mg/kg	25.21	895.70	106.68	71.26	177.41

^a^Fermented soybean meal (Industrial De Oleaginosas Americanas S.A., Barranca, Costa Rica)

**Table 2:** Ingredient composition of experimental diets (as-fed basis)

**Ingredient, %**	**Phase 1 Control**^**a**^	**Phase 2 Control**^**a**^	**Phase 3 Control**^**a**^
Corn	41.78	47.96	61.82
Soybean meal	20.00	26.00	25.00
Wheat middlings	2.50	5.00	7.50
Fermented soybean meal^b^	10.00	7.50	-
Lactose	15.00	7.50	-
Fish meal	5.00	-	-
Soybean oil	2.18	2.18	2.00
Limestone	0.80	1.00	0.94
Dicalcium phosphate	0.70	0.90	0.87
L-Lys HCl	0.45	0.40	0.37
DL-Met	0.16	0.15	0.10
L-Thr	0.13	0.11	0.10
Titanium dioxide	0.40	0.40	0.40
Salt	0.40	0.40	0.40
Vitamin-mineral premix^c^	0.50	0.50	0.50

^a^Two additional diets were formulated by adding a 1% premix with xylanase or a 1% premix with stimbiotic (i.e., combination of xylanase and xylo-oligosaccharides) to the control diet in each phase

The premix with xylanase contained 1.6 million BXU/kg of exogenous xylanase (0.1 kg xylanase concentrate containing 160 million BXU/kg was mixed with 9.9 kg of ground corn). The premix with stimbiotic contained 1.6 million BXU/kg of exogenous xylanase (0.1 kg xylanase plus xylo-oligosaccharides concentrate containing 160 million BXU/kg was mixed with 9.9 kg of ground corn). At 1% inclusion, the diet with xylanase and the diet with stimbiotic were expected to contain 16,000 BXU/kg of xylanase. BXU is the amount of enzyme that will release 0.06 micromoles of reducing sugars (xylose equivalents) from beechwood xylan per min at pH 5.3 and 50 °C

^b^Fermented soybean meal (Industrial De Oleaginosas Americanas S.A., Barranca, Costa Rica)

^c^The vitamin-micromineral premix provided the following quantities of vitamins and micro-minerals per kilogram of complete diet: vitamin A as retinyl acetate, 11,136 IU; vitamin D_3_ as cholecalciferol, 2,208 IU; vitamin E as DL-alpha-tocopheryl acetate, 66 IU; vitamin K as menadione dimethylprimidinol bisulfite, 1.42 mg; thiamin as thiamine mononitrate, 0.24 mg; riboflavin, 6.59 mg; pyridoxine as pyridoxine hydrochloride, 0.24 mg; vitamin B_12_, 0.03 mg; D-pantothenic acid as D-calcium pantothenate, 23.5 mg; niacin, 44.1 mg; folic acid, 1.59 mg; biotin, 0.44 mg; Cu, 20 mg as copper chloride; Fe, 126 mg as ferrous sulfate; I, 1.26 mg as ethylenediamine dihydriodide; Mn, 60.2 mg as manganese hydroxychloride; Se, 0.3 mg as sodium selenite and selenium yeast; and Zn, 125.1 mg as zinc hydroxychloride

**Table 3:** Analyzed nutrient composition of experimental diets (as-fed basis)

Item	Phase 1			Phase 2			Phase 3		
	Control	Xylanase^a^	Stimbiotic^b^	Control	Xylanase	Stimbiotic	Control	Xylanase	Stimbiotic
Gross energy, kcal/kg	3,997	3,997	3,982	3,881	3,892	3,907	3,833	3,849	3,887
Dry matter, %	89.66	89.61	89.40	86.80	87.45	87.22	86.52	86.30	87.03
Ash, %	6.00	6.11	5.84	4.59	4.80	5.82	5.15	5.52	5.16
Acid hydrolyzed ether extract, %	4.21	3.80	3.88	3.10	2.93	3.02	4.59	4.71	5.55
Crude protein, %	20.33	20.66	20.54	17.10	17.45	17.99	15.91	16.03	16.37
Starch, %	29.59	29.20	29.34	38.34	37.47	36.27	40.75	40.40	40.83
Insoluble dietary fiber, %	9.90	10.10	9.90	11.40	11.30	11.10	12.80	12.80	12.70
Soluble dietary fiber, %	3.30	3.40	3.30	2.80	2.50	2.30	1.90	1.80	1.40
Total dietary fiber, %	13.20	13.50	13.20	14.20	13.80	13.40	14.70	14.60	14.10
Indispensable amino acids, %									
Arg	1.25	1.23	1.26	1.08	1.12	1.21	1.00	1.03	1.04
His	0.52	0.50	0.52	0.45	0.45	0.46	0.42	0.42	0.43
Ile	0.95	0.91	0.96	0.79	0.79	0.80	0.71	0.69	0.70
Leu	1.68	1.62	1.68	1.46	1.46	1.51	1.40	1.36	1.38
Lys	1.54	1.53	1.55	1.22	1.21	1.30	1.09	1.10	1.17
Met	0.50	0.49	0.50	0.35	0.37	0.40	0.31	0.32	0.35
Phe	0.99	0.96	1.00	0.86	0.86	0.88	0.79	0.78	0.80
Thr	0.84	0.87	0.88	0.70	0.74	0.75	0.65	0.68	0.71
Trp	0.23	0.22	0.22	0.17	0.18	0.20	0.15	0.17	0.16
Val	0.98	0.98	0.98	0.85	0.84	0.90	0.79	0.76	0.77
Dispensable amino acids, %									
Ala	1.04	1.01	1.00	0.84	0.86	0.85	0.83	0.82	0.82
Asp	2.07	2.03	1.90	1.74	1.78	1.90	1.56	1.58	1.62
Cys	0.31	0.29	0.26	0.28	0.28	0.30	0.26	0.27	0.28
Glu	3.49	3.43	3.30	3.05	3.14	3.40	2.87	2.87	2.94
Gly	0.94	0.92	0.91	0.71	0.73	0.74	0.67	0.68	0.68
Pro	1.17	1.11	1.03	1.04	1.01	1.00	0.99	0.94	0.97
Ser	0.80	0.82	0.75	0.69	0.76	0.78	0.66	0.71	0.73
Tyr	0.64	0.63	0.60	0.55	0.57	0.56	0.49	0.52	0.53
Minerals									
Ca, %	2.27	1.92	2.14	1.37	1.01	1.33	1.03	0.92	0.94
P, %	1.40	1.34	1.57	1.11	1.12	1.09	0.71	0.76	0.87
K, %	1.71	1.59	1.78	1.54	1.80	1.66	0.95	0.95	1.13
Mg, %	0.31	0.28	0.31	0.29	0.33	0.29	0.21	0.20	0.23
Na, %	0.39	0.37	0.42	0.23	0.25	0.27	0.17	0.18	0.23
Cu, mg/kg	197.81	120.34	107.43	90.52	92.54	89.55	64.76	60.87	66.59
Fe, mg/kg	410.31	381.84	437.13	312.18	332.70	352.69	225.82	225.53	227.05
Mn, mg/kg	228.78	207.76	227.25	181.26	206.79	199.30	152.33	146.98	164.10
Zn, mg/kg	469.96	502.10	481.37	279.58	310.95	296.05	187.09	185.42	275.31
Xylanase activity, BXU^c^/kg	< 2,000	13,700	16,600	< 2,000	15,000	23,700	< 2,000	13,900	17,800

^a^Xylanase: Econase XT 25 (160,000 BXU/g); AB Vista, Marlborough, UK

^b^Stimbiotic: Signis; AB Vista, Marlborough, UK

^c^BXU is the amount of enzyme that will release 0.06 micromoles of reducing sugars (xylose equivalents) from beechwood xylan per min at pH 5.3 and 50 °C. None of the control diets exceeded the detection limit (2,000 BXU/kg)

### Collection of samples and data

Individual pig weights were recorded on the day of weaning, on d 14, d 28, and d 42. Feed addition to each pen was recorded daily, and the weight of feed left in the feeder was recorded on d 14, d 28, and d 42. Diarrhea scores were assessed visually per pen every other day using a score from 1 to 5 (1 = normal feces; 2 = moist feces; 3 = mild diarrhea; 4 = severe diarrhea; and 5 = watery diarrhea). Diarrhea frequency was obtained by totaling the number of pen days with diarrhea scores ≥ 3 divided by the total number of pen days multiplied by 100.

On d 14, the pig in each pen with a body weight closest to the pen average was identified (4 barrows and 4 gilts per treatment) and sacrificed via captive bolt stunning. Ileal tissue samples between 2 and 3 cm long were collected approximately 80 cm from the ileal-cecal junction. Samples were cut and pinned with the serosa side down on a piece of cardboard and then fixed in 10% neutral buffered formalin for morphological evaluation and immunohistochemistry staining. Ileal mucosa samples were washed with phosphate-buffered saline, scraped gently, snap-frozen in liquid nitrogen, and stored at −80 °C until used for ribonucleic acid (RNA) extraction.

On d 28, 2 blood samples were collected via vena puncture from 1 pig per pen. One sample was collected in a vacutainer with heparin, and the other sample was collected in a vacutainer with ethylenediaminetetraacetic acid (EDTA). Both samples were centrifuged at 2,000 × *g* at 4 °C for 15 min to yield blood plasma, which was stored at −20 °C until analyzed. Fecal samples were collected from all pigs for 3 d (d 26 to 28) in phase 2, and for 3 d (d 40 to 42) in phase 3 via anal stimulation. Collected fecal samples were stored at −20 °C until processed.

### Chemical analyses

At the conclusion of the experiment, fecal samples from each pen were thawed and dried in a 50 °C forced-air drying oven, and ground using a grain mill (model: RRH-500, Zhejiang Winki Plastic Industry Co., Ltd., Zhejiang, China) before analysis. Samples of the major ingredients used in the diets and a sample of all diets were collected at the time of diet mixing and ground before analysis. Ingredients, diets, and fecal samples were analyzed for dry matter determined by oven drying at 135 °C for 2 h (method 930.15; [18]), and diets and ingredients were also analyzed for ash (method 942.05; [18]). Acid hydrolyzed ether extract was analyzed in diets and ingredients by acid hydrolysis using 3 mol/L HCl (AnkomHCl Hydrolysis System, Ankom Technology, Macedon, NY, USA) followed by fat extraction (method 2003.06; [18]) using petroleum ether (AnkomXT-15 Extractor, Ankom Technology, Macedon, NY, USA). Diets and ingredients were also analyzed for amino acids on an amino acid analyzer (model L8800 Hitachi Amino Acid Analyzer, Hitachi High Technologies America Inc., Pleasanton, CA, USA) using ninhydrin for postcolumn derivatization and norleucine as the internal standard. Prior to analysis, samples were hydrolyzed with 6 mol/L HCl for 24 h at 110 °C (method 982.30 E(a); [18]). Methionine and Cys were determined as Met sulfone and cysteic acid after cold performic acid oxidation overnight before hydrolysis (method 982.30 E(b); [18]). Tryptophan was determined after NaOH hydrolysis for 22 h at 110 °C (method 982.30 E(c); [18]). Calcium, P, K, Mg, Na, Cu, Fe, Mn, and Zn in diets and ingredients were analyzed (method 985.01 A, B, and C; [18]) using inductively coupled plasma-optimal emission spectrometry (Avio 200, PerkinElmer, Waltham, MA, USA). Sample preparation included dry ashing at 600 °C for 4 h (method 942.05; [18],) and wet digestion with nitric acid (method 3050 B; [19]). Ingredients, diets and fecal samples were analyzed for GE determined with bomb calorimetry (Model 6400; Parr Instruments, Moline, IL, USA) using benzoic acid as the standard for calibration. Ingredients, diets, and fecal samples were analyzed for nitrogen using the combustion procedure (method 990.03; [18]) on a LECO FP628 Nitrogen Analyzer (Leco Corp., St. Joseph, MI, USA). Crude protein was calculated as 6.25 × N. Starch was analyzed in diets, ingredients, and fecal samples by the glucoamylase procedure (method 979.10; [18]). These samples were also analyzed for insoluble dietary fiber (IDF) and soluble dietary fiber (SDF) according to method 991.43 [18] using the AnkomTDF Dietary Fiber Analyzer (Ankom Technology, Macedon, NY, USA). Total dietary fiber was calculated as the sum of IDF and SDF. Fecal samples and diets were analyzed for titanium [20]. Diets were analyzed for xylanase activity using the QuantiPlate ELISA kit specific for Econase XT (ESC Standard Analytical Method SAM115; AB Vista, Plantation, FL, USA).

Heparinized plasma samples were analyzed for plasma urea N, total protein, and albumin using a Beckman Coulter Clinical Chemistry AU analyzer (Beckman Coulter, Inc., Brea, CA, USA). Plasma EDTA samples were analyzed for peptide YY (PYY) and gastric inhibitory polypeptide (GIP) using assay kits according to manufacturer specifications (Phoenix Pharmaceuticals Inc.; My BioSource, San Diego, CA, USA; respectively). The EDTA samples were also analyzed for tumor necrosis factor-alpha (TNFα), interferon-γ (IFNγ), and interleukin (IL) 1α, 1β, 1RA, 2, 4, 6, 8, 10, 12, and 18 using a cytokine/chemokine magnetic bead panel according to manufacturer specifications (MILLIPLEX Porcine Cytokine/Chemokine Magnetic Bead Panel; EMD Millipore, Darmstadt, Germany).

Morphology was measured in ileal tissue [21]. After fixation, each ileal tissue sample was cut into 2 to 3 mm thick cross-sections and embedded in paraffin for slide preparation. From each sample, three to four transverse sections were selected and stained with hematoxylin and eosin. Slides were scanned using a 2.0-HT NanoZoomer (Hamamatsu, Bridgewater, NJ, USA) and 10 intact, well-oriented villi and associated crypts were identified. Villus height, crypt depth, and lamina propria thickness of the ileum were measured of each sample in duplicate using an image processing and analysis system (NDP.View2, Hamamatsu, Bridgewater, NJ, USA). Villus height:crypt depth (VH:CD) was also calculated.

The RNA was extracted from 30 ± 0.2 mg of frozen ileal mucosa using β-mercaptoethanol (Sigma-Aldrich, St. Louis, MO, USA) according to the RNeasy Mini Kit (QIAGEN, Germantown, MD, USA) manufacturer’s instructions [21]. Total RNA was quantified using a NanoDrop ND-1000 spectrophotometer (NanoDrop Technologies, Wilmington, DE, USA). The RNA quality was determined using a Fragment AnalyzerTM Automated CE System (Method DNF-471-33-SS Total RNA 15nt; Advanced Analytical, Ankeny, IA, USA), and RNA samples with an RNA quality number greater than 7 were diluted to 100 ng/μL with nuclease-free water and used for complementary deoxyribonucleic acid (cDNA) synthesis. The cDNA was then diluted 1:4 with nuclease-free water to conduct qRT-PCR analysis which was performed using 4 µL of diluted cDNA and 6 µL of a mixture including forward and reverse primers, SYBR Green master mix (Quanta Biosciences Inc., Gaithersburg, MD, USA) and nuclease-free water in a MicroAmp^TM^ Optical 384-Well Reaction Plate (Applied Biosystems, Foster City, CA, USA). Two internal control genes, glyceraldehyde 3-phosphate dehydrogenase [22] and hypoxanthine-guanine phosphoribosyl transferase [23], were used to normalize the abundance of tested genes (Table S1). Tested genes included occludin and zonula occludens-1 because these genes regulate intestinal permeability and paracellular absorption of nutrients [24].

### Calculations and statistical analyses

At the end of the experiment, data were summarized to calculate average daily feed intake (ADFI), average daily gain (ADG), and gain:feed ratio (G:F) within each pen and treatment group. Data were summarized for d 1 to 14, d 15 to 28, d 29 to 42, and for the entire experiment. If a pig was removed from a pen during the experiment, ADFI and G:F were adjusted for the feed consumed by the pig that was removed, as described by Lindemann and Kim [25].

Apparent total tract digestibility of dry matter, GE, starch, crude protein, IDF, SDF, and TDF were calculated for each diet for phases 2 and 3, and the concentration of DE in each diet and phase was also calculated [26]. Data from the qRT-PCR analysis were analyzed using QuantStudio™ Real-time PCR software (version 1.3; Applied Biosystems, Foster City, CA), using the relative standard curve method for quantification [27].

Data were analyzed in a 2 × 3 factorial using the MIXED procedure of SAS (SAS Inst. Inc., Cary, NC, USA). Pen was the experimental unit for growth performance and digestibility of nutrients and energy, whereas the pig was the experimental unit for all markers of intestinal health. The model included sow treatment, post-weaning dietary treatment, and the interaction between sow group and post-weaning dietary treatment as fixed effects, and block and replicate within block as random effects. However, for all response parameters, no interactions were observed, therefore, the final model only included sow treatment and post-weaning dietary treatment as main effects. Normality of the residuals was confirmed, and outliers were identified using the UNIVARIATE procedure of SAS. Outliers were defined as observations with internally studentized residuals less than 3 or greater than 3, but no were identified. Least square means were calculated for each independent variable, and means were separated using the PDIFF option with Tukey adjustment [28]. The Chi-squared test was used to analyze the frequency of diarrhea among treatments. Least squares means were calculated and means were separated for the frequency of diarrhea data using GENMOD procedure of SAS. Statistical significance and tendencies were considered at *P* < 0.05 and 0.05 ≤ *P* < 0.10, respectively.

## Results

### Growth performance

There were no effects of sow diet treatment on growth performance parameters of pigs during the 42-d post-weaning period (Table 4). Phase 1 ADG, ADFI, G:F, and body weight were not affected by post-weaning dietary treatments. Phase 2 ADG and G:F were greater (*P* < 0.05) for pigs fed diets with stimbiotic compared with pigs fed the control diet or the diet with xylanase, but pigs fed the diet with xylanase had greater (*P* < 0.05) G:F than pigs fed the control diet. At the conclusion of phase 2, body weight tended to be greater (*P* < 0.10) for pigs fed the diet with stimbiotic compared with pigs fed the control diet. During phase 3, pigs fed diets with stimbiotic or xylanase had greater (*P* < 0.05) ADG compared with pigs fed the control diet, and ADFI tended to be greater (*P* < 0.10) by pigs fed the diet with stimbiotic than by pigs fed the control diet. Pigs fed a diet with stimbiotic or xylanase also tended to have greater (*P* < 0.10) G:F compared with pigs fed the control diet. For the entire experimental period, pigs fed diets with stimbiotic or xylanase had greater (*P* < 0.05) ADG, G:F, and final body weight on d 42 post-weaning than pigs fed the control diet.

**Table 4:** Growth performance of pigs^1,2^

Item	Sow treatment				Post-weaning dietary treatment				
	Control	Xylanase^3^	SEM	*P*-value	Control	Xylanase	Stimbiotic^3^	SEM	*P*-value
Phase 1 (d 1 to 14)									
Initial body weight, kg	5.77	5.84	0.20	0.589	5.81	5.83	5.77	0.21	0.924
ADG^4^, g	97.44	83.95	10.79	0.196	102.40	85.17	84.54	11.65	0.235
ADFI^4^, g	171.90	171.60	8.28	0.979	182.10	165.30	167.90	8.28	0.294
G:F^4^	0.54	0.49	0.05	0.187	0.56	0.50	0.50	0.06	0.308
Final body weight, kg	7.13	7.02	0.24	0.555	7.25	7.02	6.96	0.26	0.395
Phase 2 (d 15 to 28)									
ADG, g	430.60	440.50	15.54	0.542	391.70^b^	434.70^b^	480.20^a^	16.99	< 0.001
ADFI, g	654.20	666.40	22.11	0.632	652.10	663.80	665.10	24.60	0.885
G:F	0.66	0.66	0.01	0.867	0.60^c^	0.66^b^	0.73^a^	0.01	< 0.001
Final body weight, g	13.17	13.18	0.45	0.973	12.73^y^	13.11^xy^	13.68^x^	0.47	0.073
Phase 3 (d 29 to 42)									
ADG, g	618.10	626.10	14.95	0.701	566.80^b^	648.30^a^	651.20^a^	17.25	0.001
ADFI, g	1,054.10	1,037.40	26.90	0.577	1,001.10^y^	1,071.40^xy^	1,064.60^x^	29.73	0.084
G:F	0.59	0.61	0.02	0.310	0.57^y^	0.61^x^	0.62^x^	0.02	0.098
Final body weight, kg	21.78	21.99	0.45	0.697	20.67^b^	22.18^a^	22.80^a^	0.50	0.004
Overall (d 1 to 42)									
ADG, g	381.70	383.90	8.72	0.851	353.60^b^	389.40^a^	405.30^a^	10.04	0.001
ADFI, g	626.10	625.80	11.19	0.983	611.80	633.50	632.50	13.71	0.455
G:F	0.61	0.62	0.02	0.441	0.58^b^	0.61^a^	0.64^a^	0.02	< 0.001

^a−c^Within columns in the post-weaning dietary treatment main effect, values within a row lacking a common superscript differ (*P* < 0.05)

^x,y^Within columns in the post-weaning dietary treatment main effect, values within a row lacking a common superscript tend to be different (*P* < 0.10)

^1^Data are least square means of 24 observations per sow treatment, and 16 observations per post-weaning dietary treatment

^2^There was no interaction between sow treatment and post-weaning dietary treatment for any of the growth performance parameters, therefore, the interaction term was removed from the final model and only main effects are presented

^3^Xylanase: Econase XT 25 (160,000 BXU/g); AB Vista, Marlborough, UK; Stimbiotic: Signis; AB Vista, Marlborough, UK

^4^*ADG* Average daily gain, *ADFI* Average daily feed intake, *G:F* Gain to feed ratio

There were no differences among treatments in diarrhea scores in phases 1, 2, or overall, but during phase 3, pigs from sows fed a diet without xylanase in lactation tended to have reduced (*P* < 0.10) diarrhea scores compared with pigs from sows fed a diet with xylanase in lactation (Table 5). During phase 3, pigs fed the control diet also had lower (*P* < 0.05) diarrhea scores compared with pigs fed a diet containing stimbiotic or xylanase. There were no differences in the frequency of diarrhea by pigs from sows fed a diet without or with xylanase in phases 1, 2, or overall; however, the frequency of diarrhea during phase 1 tended to be reduced (*P* < 0.10) in pigs fed the diet with xylanase compared with pigs fed the other diets, but in phase 2, pigs fed the control diets tended to have lower (*P* < 0.10) frequency of diarrhea compared with pigs fed the other diets. During phase 3, the frequency of diarrhea was reduced (*P* < 0.05) in pigs fed the control diet compared with pigs fed the diets containing xylanase or stimbiotic.

**Table 5:** Average diarrhea scores and incidence of diarrhea in pigs^1,2^

Item	Sow treatment				Post-weaning dietary treatment				
	Control	Xylanase^3^	SEM	*P*-value	Control	Xylanase	Stimbiotic^3^	SEM	*P*-value
Diarrhea score^4^									
d 1 to 14 (Phase 1)	2.30	2.23	0.24	0.551	2.37	2.13	2.30	0.25	0.192
d 15 to 28 (Phase 2)	2.34	2.33	0.13	0.935	2.18	2.39	2.43	0.14	0.188
d 29 to 42 (Phase 3)	1.42	1.58	0.07	0.090	1.26^b^	1.71^a^	1.54^a^	0.08	0.001
d 1 to 42 (Overall phase)	2.02	2.05	0.11	0.711	1.93	2.07	2.09	0.12	0.202
Frequency of diarrhea									
d 1 to 14 (Phase 1)									
Pen days^5^	168	168	-	-	112	112	112	-	-
Frequency^6^	39.88	42.86	-	0.580	48.21	33.93	41.96	-	0.094
d 15 to 28 (Phase 2)									
Pen days	168	168	-	-	112	112	112	-	-
Frequency	38.10	41.67	-	0.504	31.25	41.96	46.43	-	0.058
d 29 to 42 (Phase 3)									
Pen days	168	168	-	-	112	112	112	-	-
Frequency	6.55	11.31	-	0.126	1.79^c^	16.96^a^	8.04^b^	-	< 0.001
d 1 to 42 (Overall phase)									
Pen days	504	504	-	-	336	336	336	-	-
Frequency	28.14	31.94	-	0.192	27.08	30.95	32.14	-	0.327

^a–c^Within columns in the post-weaning dietary treatment main effect, values within a row lacking a common superscript differ (*P* < 0.05)

^1^Data are least square means of 24 observations per sow treatment, and 16 observations per post-weaning dietary treatment

^2^There was no interaction between sow treatment and post-weaning dietary treatment for any of the diarrhea scores and incidence of diarrhea parameters, therefore, the interaction term was removed from the final model and only main effects are presented

^3^Xylanase: Econase XT 25 (160,000 BXU/g); AB Vista, Marlborough, UK; Stimbiotic: Signis; AB Vista, Marlborough, UK

^4^Fecal scores: 1 = normal feces; 2 = moist feces; 3 = mild diarrhea; 4 = severe diarrhea; and 5 = watery diarrhea

^5^Pen days = number of pens × number of day assessing diarrhea scores

^6^Frequency = (number of pen days with diarrhea scores ≥ 3 divided by pen days) × 100

### Digestibility of nutrients

No effects of sow treatment on digestibility of energy or nutrients during the 42-d post-weaning period were observed (Table 6). The ATTD of dry matter, GE, crude protein, IDF, and TDF in phase 2 was greater (*P* < 0.05) by pigs fed the diet with stimbiotic compared with pigs fed the diet with xylanase or the control diet. Likewise, the concentration of DE was greater (*P* < 0.05) in the diet with stimbiotic than in the other two diets. In phase 3, pigs fed diets with stimbiotic or xylanase had greater (*P* < 0.05) ATTD of dry matter, GE, IDF, and TDF, and greater (*P* < 0.05) DE than pigs fed the control diet. The ATTD of crude protein was also greater (*P* < 0.05) in the diet with stimbiotic than in the control diet. The ATTD of starch and SDF did not differ among treatments in phases 2 and 3.

**Table 6:** Apparent total tract digestibility (ATTD) of nutrients and digestible energy by pigs^1,^^2^

Item	Sow treatment				Post-weaning dietary treatment				
	Control	Xylanase^3^	SEM	*P*-value	Control	Xylanase	Stimbiotic^3^	SEM	*P*-value
Phase 2 (d 26 to 28)									
ATTD of dry matter, %	79.22	78.49	0.72	0.398	76.45^b^	78.42^b^	81.69^a^	0.81	< 0.001
ATTD of gross energy, %	77.83	76.74	0.84	0.260	74.50^b^	76.80^b^	80.55^a^	0.93	< 0.001
Digestible energy, kcal/kg	3,030	2,988	32.68	0.260	2,901^b^	2,990^b^	3,136^a^	32.68	< 0.001
ATTD of starch, %	98.28	98.10	0.19	0.510	97.83	98.18	98.56	0.24	0.103
ATTD of crude protein, %	74.67	73.60	1.29	0.391	71.64^b^	73.65^b^	77.11^a^	1.39	0.001
ATTD of IDF^4^, %	43.92	41.24	2.34	0.284	35.76^b^	41.24^b^	50.75^a^	2.56	< 0.001
ATTD of SDF^4^, %	76.39	79.66	1.97	0.211	75.40	77.88	80.80	2.25	0.192
ATTD of TDF^4^, %	49.89	48.28	1.99	0.451	43.04^b^	47.96^b^	56.26^a^	2.18	< 0.001
Phase 3 (d 40 to 42)									
ATTD of dry matter, %	79.57	78.97	0.53	0.376	77.70^b^	79.69^a^	80.42^a^	0.60	0.002
ATTD of gross energy, %	78.26	77.64	0.56	0.388	76.16^b^	78.53^a^	79.15^a^	0.64	0.001
Digestible energy, kcal/kg	3,018	2,994	21.63	0.388	2,937^b^	3,028^a^	3,052^a^	24.51	0.001
ATTD of starch, %	98.55	98.48	0.14	0.727	98.30	98.66	98.59	0.17	0.286
ATTD of crude protein, %	74.70	73.24	0.75	0.173	72.32^b^	73.94^ab^	75.65^a^	0.88	0.030
ATTD of IDF, %	49.39	47.28	1.56	0.243	44.06^b^	49.63^a^	51.32^a^	1.74	0.002
ATTD of SDF, %	66.35	67.33	4.60	0.807	67.06	66.61	66.85	4.90	0.995
ATTD of TDF, %	51.48	49.55	1.80	0.246	46.76^b^	51.63^a^	53.15^a^	1.93	0.004

^a,b^Within columns in the post-weaning dietary treatment main effect, values within a row lacking a common superscript differ (*P* < 0.05)

^1^Data are least square means of 24 observations per sow treatment, and 16 observations per post-weaning dietary treatment

^2^There was no interaction between sow treatment and post-weaning dietary treatment for any of the digestibility parameters, therefore, the interaction term was removed from the final model and only main effects are presented

^3^Xylanase: Econase XT 25 (160,000 BXU/g); AB Vista, Marlborough, UK; Stimbiotic: Signis; AB Vista, Marlborough, UK

^4^*IDF* Insoluble dietary fiber, *SDF* Soluble dietary fiber, *TDF* Total dietary fiber

### Markers of intestinal health

In phase 2, pigs fed the diet with xylanase tended to have reduced (*P* < 0.10) plasma urea N compared with pigs fed the diet with stimbiotic (Table 7). Plasma total protein concentration was reduced (*P* < 0.05) in pigs from sows fed the diet with xylanase in lactation compared with pigs from sows fed a diet without xylanase, but pigs fed the diet with stimbiotic had greater (*P* < 0.05) plasma total protein and albumin than pigs fed the control diet. Pigs fed the diet with xylanase had reduced (*P* < 0.05) IL-1α, IL-2, IL-4, and IL-10 in plasma, and tended to have reduced (*P* < 0.10) IL-1β compared with pigs fed the other diets. However, pigs from sows fed xylanase in lactation had reduced (*P* < 0.05) IL-1RA and tended to have reduced (*P* < 0.10) IL-8 compared with pigs from sows fed a diet without xylanase. No differences were observed in villus height, crypt depth, lamina propria thickness, VH:CD, or mRNA abundance of occludin and zonula occludens-1 among treatment groups at the end of phase 1 (Tables 8 and 9).

**Table 7:** Plasma characteristics and cytokines of pigs (d 28)^1,2^

Item	Sow treatment				Post-weaning dietary treatment				
	Control	Xylanase^3^	SEM	*P*-value	Control	Xylanase	Stimbiotic^3^	SEM	*P*-value
Plasma urea N, mg/dL	6.00	5.96	0.42	0.944	6.25^xy^	5.00^y^	6.69^x^	0.51	0.062
Total protein, g/dL	5.00	4.81	0.04	0.002	4.81^b^	4.89^ab^	5.02^a^	0.05	0.020
Albumin, g/dL	2.90	2.88	0.08	0.805	2.77^b^	2.91^ab^	2.99^a^	0.08	0.012
Peptide YY, ng/mL	1.24	1.35	0.06	0.162	1.31	1.31	1.27	0.07	0.914
GIP^4^, pg/mL	788.50	839.53	68.61	0.545	797.26	741.32	903.47	75.25	0.203
Cytokine, ng/mL									
IFNγ^4^	4.62	4.85	1.04	0.796	5.86	3.61	4.75	1.12	0.123
IL^4^-1α	0.02	0.02	0.01	0.333	0.02^a^	0.01^b^	0.03^a^	0.01	0.011
IL-1β	0.10	0.09	0.01	0.345	0.11^x^	0.06^y^	0.11^x^	0.02	0.051
IL-1RA	0.28	0.18	0.02	0.004	0.24	0.19	0.26	0.03	0.185
IL-2	0.09	0.07	0.02	0.663	0.11^a^	0.03^b^	0.10^a^	0.02	0.027
IL-4	0.25	0.23	0.06	0.862	0.34^a^	0.07^b^	0.31^a^	0.07	0.007
IL-6	0.05	0.03	0.01	0.200	0.05	0.04	0.04	0.01	0.765
IL-8	0.02	0.01	0.00	0.054	0.01	0.02	0.01	0.00	0.513
IL-10	0.32	0.29	0.08	0.672	0.37^a^	0.13^b^	0.41^a^	0.09	0.010
IL-12	0.78	0.74	0.05	0.640	0.71	0.86	0.71	0.06	0.133
IL-18	0.98	0.78	0.13	0.283	0.89	0.77	0.99	0.16	0.638
TNFα^4^	0.05	0.03	0.01	0.103	0.05	0.04	0.04	0.01	0.362

^a,b^Within columns in the post-weaning dietary treatment main effect, values within a row lacking a common superscript differ (*P* < 0.05)

^x,y^Within columns in the post-weaning dietary treatment main effect, values within a row lacking a common superscript tend to be different (*P* < 0.10)

^1^Data are least square means for each dependent variable represent 18 to 24 observations per sow treatment, and 13 to 16 observations per post-weaning dietary treatment

^2^There was no interaction between sow treatment and post-weaning dietary treatment for any of the plasma and cytokines parameters, therefore, the interaction term was removed from the final model and only main effects are presented

^3^Xylanase: Econase XT 25 (160,000 BXU/g); AB Vista, Marlborough, UK; Stimbiotic: Signis; AB Vista, Marlborough, UK

^4^*GIP* Gastric inhibitory polypeptide, *IFNγ* Interferon-γ, *IL* Interleukin, *TNFα* Tumor necrosis factor-α

**Table 8:** Intestinal tissue morphology of pigs (d 14)^a,b^

Item	Sow treatment				Post-weaning dietary treatment				
	Control	Xylanase^c^	SEM	P-value	Control	Xylanase	Stimbiotic^c^	SEM	*P*-value
Ileum, μm									
Villus height	321.43	314.52	17.17	0.568	329.02	305.47	319.43	17.92	0.229
Crypt depth	238.77	237.84	12.54	0.903	241.16	234.93	238.81	12.98	0.769
Lamina propria thickness	50.18	49.11	1.13	0.509	48.46	50.83	49.64	1.39	0.493
VH:CD^d^	1.33	1.33	0.02	0.937	1.36	1.31	1.32	0.03	0.370

^a^Data are least square means for each dependent variable represent 20 to 24 observations per sow treatment, and 14 to 16 observations per post-weaning dietary treatment

^b^There was no interaction between sow treatment and post-weaning dietary treatment for any of the morphology parameters, therefore, the interaction term was removed from the final model and only main effects are shown

^c^Xylanase: Econase XT 25 (160,000 BXU/g); AB Vista, Marlborough, UK; Stimbiotic: Signis; AB Vista, Marlborough, UK

^d^VH:CD = villus height:crypt depth ratio

**Table 9:** Least squares means (log2-backtransformed) for mRNA abundance of genes in the ileum of pigs (d 14)^a,b^

Item	Sow treatment				Post-weaning dietary treatment				
	Control	Xylanase^c^	SEM	*P-*value	Control	Xylanase	Stimbiotic^c^	SEM	*P*-value
Occludin	1.10	0.88	1.12	0.147	1.00	0.90	1.06	1.14	0.666
Zonula occludens- 1	2.13	1.91	1.12	0.443	2.13	2.03	1.89	1.13	0.773

^a^Data are least square means for each dependent variable represent 20 to 24 observations per sow treatment, and 14 to 16 observations per post-weaning dietary treatment

^b^There was no interaction between sow treatment and post-weaning dietary treatment for any of the gene expression parameters, therefore, the interaction term was removed from the final model and only main effects are presented

^c^Xylanase: Econase XT 25 (160,000 BXU/g); AB Vista, Marlborough, UK; Stimbiotic: Signis; AB Vista, Marlborough, UK

## Discussion

### Ingredients and diets composition

Concentrations of dry matter, crude protein, amino acids, GE, starch, IDF, SDF, ash, and acid-hydrolyzed ether extract of ingredients were in agreement with reported values [16]. Most fiber in corn and wheat middlings consists of arabinoxylans [1], which is poorly fermented by pigs; however, exogenous xylanase hydrolyzes the β-(1−4) glycosidic bonds in arabinoxylans, releasing oligosaccharides and pentoses that potentially can be fermented by hindgut microbes with subsequent synthesis of volatile fatty acids that can be used by pigs [29]. Therefore, the ingredients used in this experiment provided the substrate for the xylanase enzyme supplemented alone or in combination with XOS. The xylanase activity for the control diets did not exceed the detection limit (2,000 BXU/kg), and the average xylanase activity for the diets with xylanase (14,200 ± 700 BXU/kg) and stimbiotic (19,370 ± 3,800 BXU/kg) was in agreement with the expected values. Likewise, the analyzed xylanase activity of the sow lactation diets was in agreement with calculated values [15].

### Growth performance and nutrient digestibility

Diets rich in dietary fiber generally have lower nutritive value and sometimes this results in reduced growth performance of weanling pigs [30]. Incorporating exogenous enzymes and prebiotics in the diet of pigs may increase nutrient digestion and absorption, resulting in enhanced growth performance [4, 9, 11].

The lack of effects of xylanase and stimbiotic on growth performance during phase 1 of this experiment was in agreement with data from experiments using grading levels of XOS [10, 11], but in disagreement with data reporting greater ADG when XOS were added to the diets from d 1 to 14 after weaning [31]. This discrepancy may have been due to differences in the xylanase and stimbiotic that were used, the doses or activity of the products, or the composition of the diets. It is also possible that animals needed to adapt to the enzyme and the stimbiotic [32], or needed to recover from post-weaning stress [33, 34].

The improved ATTD of dry matter, crude protein, IDF, TDF, and GE, and greater concentration of DE in response to addition of xylanase or stimbiotic to the diets is in agreement with data indicating that addition of xylanase to diets for broiler chickens or pigs increased digestibility of dry matter, crude protein, energy, and non-starch polysaccharides [4, 5, 35–38]. Xylanase hydrolyzes the xylose backbone of arabinoxylans, which may result not only in greater solubilization of insoluble dietary fiber and thus available to fermentation, but also in the release of trapped nutrients, and reduced digesta viscosity, leading to an increase in nutrient and energy digestibility [39–44]. However, it has not been demonstrated that changes in viscosity due to addition of xylanase to diets for pigs have any impact on nutrient digestibility [4]. Likewise, XOS are sugar oligomers (2 to 10 monomeric units), which may act as prebiotics in the diet for pigs, modifying the microbial ecology [45, 46], resulting in increased activity of beneficial gut bacteria such as *Bifidobacterium* spp. [47], reduced pH in the hindgut, and increased synthesis of short-chain fatty acids that decrease intestinal pathogens and provide energy to the host [10, 48–50]. Because of the modes of action of xylanases and stimbiotics, these additives are believed to be nutrient enhancing compounds that have the overall effects of increasing energy absorption by pigs. Results of the experiment demonstrating the increased ATTD of fiber and energy support this hypothesis. Improvements in digestibility of dry matter, protein, energy, and concentrations of DE observed during phases 2 and 3 as xylanase or stimbiotic was included in the diets resulted in an improvement in growth performance, which is likely primarily due to increased hydrolysis of insoluble dietary fiber. It is possible that the combination of the mechanisms of action of xylanase and XOS increased the efficiency of fermenting dietary fiber as indicated by the greater effect of adding stimbiotic than xylanase to the phase-2 diets. As a consequence, the hypothesis that xylanase and stimbiotic improve growth performance and energy and fiber digestibility when included in diets for weanlings pigs was accepted.

The observation that sow treatment had no impact on growth performance or nutrient digestibility by the offspring indicates that there is no carry over effect from sows to the offspring. This may indicate that xylanase in sow diets does not change the microbial composition of sow feces, but because we did not determine microbial composition, we cannot confirm this hypothesis.

### Diarrhea incidence and markers of intestinal health

The observed increase in diarrhea incidence for pigs fed xylanase in phase 3 is in contrast with data indicating that xylanase reduced diarrhea rate or did not impact diarrhea [37, 51, 52], and XOS reduced diarrhea rate compared with xylanase when fed to pigs [53, 54]. The type of dietary fiber (soluble vs. insoluble) may influence the physicochemical properties of the digesta and the intestinal microbiota [55], which may be impacted by xylanase or stimbiotic supplementation. More research needs to be conducted to clarify the effects of xylanase and stimbiotic on diarrhea in weanling pigs. However, it is possible that xylanase resulted in some insoluble fiber becoming solubilized, and if these soluble fibers were not fully fermented in the hindgut, they may have resulted in higher moisture content and loss of fecal consistency.

Low plasma urea N is an indicator of efficient amino acid utilization [56]. The observation that pigs fed the diet with xylanase tended to have reduced plasma urea N was in agreement with decreased plasma urea N concentration when xylanase was fed to weanling pigs [37]. Total protein and albumin are indicators of transport of nutrients in the blood [57] and the increased total protein and albumin observed in pigs fed diets containing stimbiotic in phase 2, therefore, indicates that nutrient transport was more efficient, which may have supported the increased growth performance.

Peptide YY and GIP are gastrointestinal hormones that are important in the regulation of feed intake, energy homeostasis, gastric acid secretion, and gastrointestinal motility, and their levels increase immediately after nutrient ingestion [58, 59]. It was hypothesized that PYY and GIP would be impacted by xylanase and stimbiotic, as previously reported [5, 60], but the lack of effects of both xylanase and stimbiotic on stimulating PYY and GIP indicates that factors other than those determined in this experiment influence plasma concentrations of PYY and GIP.

Fibrous ingredients fed to weanling pigs may increase oxidative stress and intestinal inflammation due to physicochemical characteristics resulting in increased digesta viscosity, and greater pathogenic load [7]. Inflammation is regulated by the immune system by innate and adaptive responses, which produce cytokines with pro-inflammatory or anti-inflammatory effects [61]. The reduction in the pro-inflammatory cytokines IL-1α and IL-2 and the anti-inflammatory cytokines IL-4 and IL-10 indicates a positive effect of xylanase on the immune response of pigs, possibly due to alteration of the intestinal microbiota composition. The lack of an effect of stimbiotic on concentrations of cytokines is in contrast with results of research indicating that there are anti-inflammatory effects of stimbiotic in pigs and broilers [62, 63]. This discrepancy indicates that stimbiotic may interact with the gut microbiota differently than xylanase alone, but we cannot confirm this hypothesis because we did not determine microbial composition. The impact of xylanase and stimbiotic on immune response may also depend on the existing microbial community [64], which can vary among environmental conditions, pig genetics, health status, and diet composition. The potential mechanism of xylanase and stimbiotic associated with the reduction in inflammatory response parameters remains unclear and research to elucidate effects of xylanase and stimbiotic on regulation of cytokines is needed.

An improved nutrient digestibility in diets containing xylanase or stimbiotic may result from changes in intestinal morphology because intestinal villi are the site of nutrient absorption [4, 5, 65]. However, the lack of any impact of xylanase or stimbiotic on intestinal morphology, which has also been observed previously [6, 31], indicates that the increased ATTD of energy and nutrients that was observed for pigs fed xylanase or stimbiotic was unrelated to changes in intestinal morphology.

The permeable barrier of the intestine may be modified by xylanase or stimbiotic added to diets for pigs, resulting in increased relative mRNA abundance of tight junction proteins (i.e., occludin and zonula occludens-1) in the jejunum and ileal mucosa of pigs [5–7, 66]. However, the observation that xylanase or stimbiotic did not impact the mRNA abundance of tight junction proteins in this experiment, despite greater nutrient digestibility, indicates that there may be other interactions among diet, mucosa, and the intestinal microbiota that may impact intestinal integrity.

## Conclusions

Feeding diets with xylanase or stimbiotic to weanling pigs increased nutrient digestibility in the late nursery, leading to greater growth performance after 42 d post-weaning; however, feeding sows xylanase in lactation did not influence pig growth performance after weaning. Although supplementation of a stimbiotic increased nutrient transport proteins in the blood, and xylanase led to positive immune responses by decreasing cytokines related to inflammation, xylanase or stimbiotic did not impact intestinal morphology and tight junction protein expression, which suggests that the mechanisms underlying improved digestibility and performance of weanling pigs may be more complex and involve interactions among diet, host and gut microbiota, physiology, and immune function. Additional research is needed to elucidate more biological mechanisms and interactions between host metabolism, fermentation by microbial populations, and xylanase, xylo-oligosaccharides, and stimbiotic alone or in combination in diets for weanling pigs.

## Supplementary Information

Additional file 1: Table S1. Forward and reverse primer sequences used for the quantitative reverse transcription-polymerase chain reaction.

## Data Availability

The datasets used and/or analyzed during the current study are available from the corresponding author upon reasonable request.

## Abbreviations

ADFI: Average daily feed intake
ADG: Average daily gain
ATTD: Apparent total tract digestibility
BXU: Beechwood xylanase units
cDNA: Complementary deoxyribonucleic acid
DE: Digestible energy
EDTA: Ethylenediaminetetraacetic acid
G:F: Gain to feed ratio
GE: Gross energy
GIP: Gastric inhibitory polypeptide
IDF: Insoluble dietary fiber
IFN-γ: Interferon-gamma
IL: Interleukin
PYY: Peptide YY
qRT-PCR: Quantitative reverse-transcription polymerase chain reaction
RNA: Ribonucleic acid
SDF: Soluble dietary fiber
TDF: Total dietary fiber
TNFα: Tumor necrosis factor alpha
VH:CD: Villus height:crypt depth ratio
XOS: Xylo-oligosaccharides
