# Supplementary material for: Growth performance and total tract digestibility of nutrients for weanling pigs are improved by an exogenous xylanase and a stimbiotic regardless of maternal xylanase consumption
Source: J Anim Sci Biotechnol. 2025 May 15;16:68. doi: 10.1186/s40104-025-01205-w (PMC12080015; doi:10.1186/s40104-025-01205-w)
Supplement: Supplementary file 1 — Additional file 1: Table S1. Forward and reverse primer sequences used for the quantitative reverse transcription-polymerase chain reaction. [file 40104_2025_1205_MOESM1_ESM.docx]

**Table S1.** Forward and reverse primer sequences used for the quantitative reverse transcription-polymerase chain reaction

|  | Primer sequences (5’→3’) | |  |
| --- | --- | --- | --- |
| Item | Forward | Reverse | Reference |
| Internal control genes | | | |
| HPRT^1^ | GGACTTGAATCATGTTTGTG | CAGATGTTTCCAAACTCAAC | [1] |
| GAPDH^1^ | ATCCTGGGCTACACTGAGGAC | AAGTGGTCGTTGAGGGCAATG | [2] |
| Gut-protective target genes | | | |
| OCLN^1^ | TCCTGGGTGTGATGGTGTTC | CGTAGAGTCCAGTCACCGCA | [3] |
| ZO1^1^ | AAGCCCTAAGTTCAATCACAATCT | ATCAAACTCAGGAGGCGGC | [3] |

^1^HPRT = Hypoxanthine-guanine phosphoribosyl transferase; GAPDH = Glyceraldehyde 3-phosphate dehydrogenase; OCLN = Occludin; ZO1 = Zonula occludens-1.

**References:**

1. Nygard AB, Jorgensen CB, Cirera S, Fredholm M. Selection of reference genes for gene expression studies in pig tissues using SYBR green qPCR. BMC Mol Biol. 2007;8:67. doi:10.1186/1471-2199-8-67.
2. Gonzalez LM, Williamson I, Piedrahita JA, Blikslager AT, Magness ST. Cell lineage identification and stem cell culture in a porcine model for the study of intestinal epithelial regeneration. PLoS One. 2013;8:e66465. doi:10.1371/journal.pone.0066465.

3. Hu CH, Xiao K, Luan ZS, Song J. Early weaning increases intestinal permeability, alters expression of cytokine and tight junction proteins, and activates mitogen-activated protein kinases in pigs. J Anim Sci. 2013;91:1094–1101. doi:10.2527/jas.2012-5796.
